# Use of imaging-based dosimetry for personalising radiopharmaceutical therapy of cancer

**DOI:** 10.1186/s40644-022-00505-y

**Published:** 2022-12-09

**Authors:** Jean-Mathieu Beauregard

**Affiliations:** 1 Department of Medical Imaging, and Research Center (Oncology Axis), CHU de Québec – Université Laval, 11 côte du Palais, Quebec City, G1R 2J6 Canada; 2 Department of Radiology and Nuclear Medicine, and Cancer Research Center, Université Laval, Quebec City, Canada

## Abstract

Theranostics – i.e., the combination of molecular imaging and radiopharmaceutical therapy of cancer targeting a common biological feature – is a rapidly expanding field owing the recent successes of novel radiopharmaceutical therapies, such as ^177^Lu-based prostate-specific membrane antigen radioligand therapy of prostate cancer and peptide receptor radionuclide therapy of neuroendocrine tumours. Despite the ongoing technical developments in imaging-based dosimetry, the existence of tumour absorbed dose-efficacy and organ absorbed dose-toxicity relationships, as well as the high interpatient variability in absorbed doses per unit activity, radiopharmaceutical therapies are still mostly administered in a fixed-activity, one-size-fits-all fashion. This is at odds with the principles of radiation oncology, where the absorbed doses to tissues are prescribed and their delivery is carefully planned and controlled for each individual patient to maximise the clinical benefits. There is a growing body of clinical evidence that dosimetry-based radiopharmaceutical therapy allows to safely optimise tumour irradiation, which translates into improved clinical outcomes. In this narrative review, we will present the reported prospective clinical experience to date on the use of imaging-based dosimetry to personalise radiopharmaceutical therapies.

Radiopharmaceutical therapy (RPT) is currently revolutionising oncological care. It consists in the administration of particle-emitting radiopharmaceuticals that are designed to specifically target the cancer lesions while sparing, as much as possible, healthy tissues. The goal of RPT is to deliver a substantial amount of radiation to the tumour to cure it or, failing that, to achieve prolonged disease control and symptomatic palliation. Most RPT applications fall under the paradigm of *theranostics* (contraction of *thera*py and diag*nostics*), in which companion diagnostic radiopharmaceuticals labelled with photon- or position-emitting radionuclides are used in conjunction with scintigraphy (including single-photon computed emission tomography, SPECT) or position emission tomography (PET), respectively, to select the patients for the RPT following demonstration of sufficient tumour uptake, which in turn is predictive of effective targeting of cancer during subsequent RPT. For this, RPT distinguishes itself from most other cancer drugs (including chemotherapy, immunotherapy, and targeted therapies), which are often given blind to disease- or patient-specific information. Exceptions include patient selection based on, e.g., germline genetic testing (e.g. BRCA mutations for poly(ADP-ribose) polymerase inhibitors) or histopathological features obtained from single-lesion biopsy, but such results provide little information on how effectively each lesion will be targeted by the drug, particularly in the setting of an heterogeneous metastatic disease.

Another aspect making RPT standing apart from other systemic modalities is that its active ingredient is not the pharmaceutical molecule itself, but the ionising radiation and consequent reactive oxygen species that are formed in situ following each decay, as well as direct DNA damage. Accordingly, RPT is a radiotherapeutic modality that is *internal* (as opposed to *external*, when x-rays or sealed sources are used) and targeted via a specific biological or mechanical pathway. The quantity of radioactivity administered is measured in becquerels (Bq; often mistakenly referred to as “dose”), whereas the absorbed dose (to which “dose” refers to in this text) is the concentration of energy deposited in tissues expressed in grays (Gy). In radiotherapy, it is the absorbed dose, fractionation, type of radiation, and the dose rate that drive the biological effects, and these variables can be integrated to some extent into metrics of biological effectiveness (e.g. biologically effective dose, BED).

RPT was born about 80 years ago with the introduction of ^32^P to treat leukaemia and of ^131^I to treat thyroid cancer. Until the turn of the century, the practice of RPT has remained limited both in terms of RPTs available and number of patients treated. However, owing to the recent successes of a new generation of ^177^Lu-based RPTs, namely peptide receptor radionuclide therapy (PRRT) of neuroendocrine tumours and prostate-specific membrane antigen radioligand therapy (PSMA-RLT) of prostate cancer, the field of theranostics is now developing at an accelerated pace and transforming the practice of nuclear medicine [1–3]. It is noteworthy that, despite these exciting advances in theranostics, the leading RPTs are still administered in an empiric fashion, i.e. with a fixed administered activity per treatment to all patients. For example, a round number of mCi – namely 200 mCi (7.4 GBq) – that is popular for ^131^I RPT of metastatic thyroid cancer, has been adopted for ^177^Lu RPTs as a convenient one-size-fits-all portion with little dosimetry rationale. This approach ignores a founding principle of radiotherapy, which is to control and standardise the delivery of radiation to tissues, i.e. absorbed doses, as opposed to administered activity. Because the specific absorbed doses (i.e., Gy per GBq administered) are highly variable across patients for any healthy organ (typically by around one order of magnitude or more), and even more so for tumours (by at least two orders of magnitude), a fixed-activity treatment is unlikely to be optimal for all. And when an empiric RPT regime is tuned towards good tolerability, this will translate into the majority of patients being undertreated, i.e. receiving less radioactivity thus less tumour irradiation than they could realistically tolerate, with the consequent risk of suboptimal outcomes. In this respect, RPT is lagging far behind external radiation therapy, where the treatments are carefully tailored to each patient’s needs.

There is a growing body of evidence supporting the personalisation of RPT based on individualised dosimetry. In this narrative review, we will summarise the current clinical experience with imaged-based dosimetry-guided protocols by highlighting the results of prospective clinical trials that are setting the bases of personalised RPT regimes. Key features and results of presented studies are compiled in Table 1.

**Table 1:** Summary of prospective clinical trials of personalised RPT studies

Reference	Radiopharmaceutical Therapy	Population	Prescription	Dosimetry	Efficacy*	Toxicity (Grade 3–4 and Serious)
Garin et al. [4] Multicentre, France	^90^Y glass microspheres radioembolisation	60 patients with hepatocellular carcinoma, **randomised**	**Personalised arm:** 205–250 Gy to the tumour ≤ 120 Gy to the normal liver **Standard arm:** 120 Gy to the perfused liver Single administration Personalised activity based on pre-treatment dosimetry	Pre-treatment ^99m^Tc-macro-aggregated albumin Planar for lung shunt SPECT/CT for tumour and liver dosimetry	**ORR: 71% vs. 36% (*p*** **= 0.0074; primary endpoint)** PFS: 6.0 vs. 3.4 mo. (*p* = 0.26). OS: 26.6 vs. 10.7 mo. (*p* = 0.0096)	Grade ≥ 3: 60% vs. 76% Serious adverse events: 20% vs. 33%
Fisher et al. [5] Multicentre, United States	^131^I-tositumomab radioimmunotherapy	250 patients with refractory/relapsed non-Hodgkin lymphoma	0.75 Gy to the whole-body or 0.65 Gy if platelet counts 100-150 × 10^− 3^/mm^3^ Single administration Personalised activity based on pre-treatment dosimetry	Pre-treatment with ^131^I-tositumomab 3-timepoint WB planar Whole-body segmentation	ORR: 56% CR: 30% PFS: 6.4 mo.	From Horning et al. [6]*:* Hematologic: 50% Thrombopenia: 25% Neutropenia: 43% Anaemia: 10% Non-hematologic: 16% From Bennett et al. [7]*:* MDS/AML: 2.3%
Kaminski et al. [8] Ann Harbor, MI, United States	^131^I-tositumomab radioimmunotherapy	76 treatment-naïve patients with stage III/IV follicular lymphoma	0.75 Gy to the whole-body Single administration Personalised activity based on pre-treatment dosimetry	Pre-treatment with ^131^I-tositumomab 3-timepoint WB planar Whole-body segmentation	ORR: 95% CR: 75% PFS: 6.1 yr.	Thrombopenia: 17% Neutropenia: 34% Anaemia: 0% Non-hematologic: 21% MDS/AML: 0%
Wahl et al. [9] Baltimore, MD, and Madison, WI, United States	^90^Y-ibritumomab myeloablative radioimmunotherapy	18 patients with refractory/relapsed non-Hodgkin lymphoma	Dose escalation to the liver, from 18 to 30.5 Gy Single administration Personalised activity based on pre-treatment dosimetry	Pre-treatment with ^111^In-ibritumomab Hybrid 5-timepoint WB planar and single SPECT/CT Whole liver segmentation	ORR: 89% CR: 72% PFS: > 13 mo.	Myeloablation expected Febrile neutropenia: 22% Hepatic: 0% MDS: 6%
Cameron et al. [10] Fremantle, Australia	^153^Sm-EDTMP RPT	10 patients with painful bone metastases	2 Gy to the bone marrow Single administration Personalised activity based on pre-treatment dosimetry	Pre-treatment with ^153^Sm-EDTMP 2-timepoint WB planar	Pain relief rate (primary): 80%	No grade 3–4 toxicity
Pryma et al. [11] Multicentre, United States	^131^I-MIBG RPT	68 patients with pheochromocytoma or paraganglioma	Fixed activity of 37 GBq (296 MBq/kg if < 62.5 kg) divided in 2 cycles, personalised (reduced) not to exceed: 12 Gy to bone marrow 16.5 Gy to lungs 18 Gy to kidneys 31 Gy to liver 40 Gy to small intestine	Pre-treatment with ^131^I-MIBG 3-timepoint WB planar Whole organ segmentation	Anti-hypertensive drugs reduction rate (primary): 25% DCR: 92% ORR: 23% OS: 37 mo.	Hematologic: 72% Thrombopenia: 41% Leucopoenia 41% Neutropenia: 38% Anaemia: 21% MDS/AML/ALL: 7%
Garske-Román et al. [12] Uppsala, Sweden	^177^Lu-DOTATATE PRRT	200 patients with mixed neuroendocrine tumours	23 Gy to the kidney Variable number of cycles 7.4 GBq/cycle	Post-treatment 3-timepoint SPECT/CT 4-cc VOI renal sampling	DCR: 97% ORR: 24% PFS: 27 mo. OS: 43 mo.	Hematologic: 15% AML/ALL: 2% Nephrotoxicity: 0.5%
Sundlöv et al. [13] Lund and Guthenberg, Sweden	^177^Lu-DOTATATE PRRT	96 patients with mixed neuroendocrine tumours	27 Gy renal BED (up to 40 Gy BED in a subset of patients) Variable number of cycles 7.4 GBq/cycle	Post-treatment Hybrid 5-timepoint planar and one-timepoint SPECT/CT Whole-kidney segmentation	DCR: 82% ORR: 16% PFS: 29 mo. OS: 47 mo.	Thrombopenia: 9% Leucopoenia: 4% Neutropenia: 6% Anaemia: 1% Nephrotoxicity: 0%
Del Prete et al. [14] Quebec City, Canada	^177^Lu-DOTATATE PRRT	54 patients with mixed neuroendocrine tumours	23 Gy to the kidney Four cycles Personalised activity at all cycles based on BSA and eGFR at 1st cycle, then post-treatment dosimetry	Post-treatment 3-timepoint SPECT/CT 4-cc VOI renal sampling	DCR: 92% ORR: 23% PFS: 16 mo. OS: not reached	Thrombopenia: 6% Leucopoenia: 6% Neutropenia: 4% Anaemia: 8% Nephrotoxicity: 0%
Menda et al. [15] Iowa City, IA, United States	^90^Y-DOTATOC PRRT	25 patents with mixed neuroendocrine tumours	23 Gy to the kidney or 2 Gy to the bone marrow 3 cycles 4.4 GBq at first cycle Personalised activity at cycles 2 and 3 based on post-treatment dosimetry	Post-treatment PET/CT at 5 h and 4-timepoint SPECT/CT Whole kidney segmentation Blood sampling for bone marrow	Not reported	Thrombopenia: 0% Neutropenia: 0% Nephrotoxicity: 0%
Bushnell et al. [16] Iowa City, IA, United States	Combined ^90^Y-DOTATOC PRRT and ^131^I-MIBG RPT	3 patients with midgut neuroendocrine tumours	19 Gy to the kidney and 1.5 Gy to the bone marrow 2 cycles Personalised activity based on pre-treatment dosimetry	Pre-treatment with ^111^In-octreotide and ^131^I-MIBG 4-timepoint planar and SPECT/CT imaging Whole kidney segmentation Blood sampling for bone marrow	PFS: 0% DCR: 100%	Thrombocytopenia: 33% (1 patient)

* Median values are reported

## ^90^Y radioembolisation of hepatocellular carcinoma

Intra-arterial administration of radiolabelled microspheres, i.e. radioembolisation, is a loco-regional modality used in the management of liver malignancy. While it can be applied to treat metastatic liver lesions such as those from colorectal cancer or neuroendocrine tumours, it has primarily been developed as a modality against inoperable primary liver cancer. Benefits of a dosimetry-based approach in radioembolisation have been clearly shown by a French group led by Garin [4]. The DOSISPHERE-01 study was a randomised, multicentre, phase 2 trial of personalised vs. standard dosimetry ^90^Y glass microsphere radioembolisation in 60 patients with hepatocellular carcinoma. The treatment planning is done using pre-treatment angiography and ^99m^Tc macroaggregated albumin (as the diagnostic surrogate tracer) planar and SPECT/CT scans to determine the volume of perfused liver and lung shunt (to limit the lung dose to 30 Gy). In the standard regime, the activity is prescribed with the assumption that it will distribute uniformly within the perfused liver volume, a worse-case scenario, with a limit of 150 Gy (target of 120 Gy) to that volume. However, this conservative approach ignores a fundamental principle of radioembolisation, i.e. that the arterial hepatic blood flow preferentially feeds the hypervascularised tumours rather than the normal liver parenchyma, which is perfused mostly by the portal vein. Accordingly, higher tumour sequestration of activity will inevitably reduce the dose to the normal liver by virtue of a sink effect. In the personalised arm, the activity prescription was escalated to deliver at least 205 Gy (target of 250 Gy) to the index tumour lesion, while not exceeding 120 Gy to the normal perfused liver parenchyma.

The prescribed median activity was significantly greater in the personalised group as compared with the standard group (3.6 vs. 2.6 GBq, respectively; *p* = 0.0049). There was a significant increase in objective response rate (ORR) of the index lesion (which was a lesion ≥7 cm) among the 28 evaluable patients in each group: 71% in the personalised group vs. 36% in the standard group (*p* = 0.0074). This constituted a statistically positive result for the primary endpoint at the interim analysis, so the trial was stopped. As a secondary endpoint, a significant difference in the median overall survival (OS) was found: 26.6 (95% CI: 11.7 – NR) mo. vs. 10.7 (95% CI: 6.0–16.8) mo., respectively (HR 0.421; 95% CI:0.215–0.826; *p* = 0.0096). Progression-free survival (PFS) was not statistically different between groups (median 6.0 vs. 3.4 mo., respectively; *p* = 0.26). In the safety analysis, at least one serious adverse event was reported in 20% and 33% of patients receiving the personalised and the standard treatments, respectively, suggesting that the personalised treatment is as well tolerated as the standard approach despite the higher administered activity. Excluding lymphopenia, grade 3 or more adverse events were mostly of hepatic or gastrointestinal nature. One treatment-related death was recorded in each group. The DOSISPHERE-01 trial results probably represent to date the most compelling evidence of the superiority of a dosimetry-based regime over an empiric one.

## ^131^I-tositumomab radioimmunotherapy of lymphoma

Radioimmunotherapy of lymphoma using an anti-CD20 radiopharmaceutical, ^131^I-tositumomab (Bexxar®), was developed as an imaging dosimetry-based RPT for patients with non-Hodgkin lymphoma. An absorbed dose escalation study was conducted and 0.75 Gy to the whole-body was found to be the maximum tolerated dose [17]. The data from five clinical trials were compiled and led to the FDA approval of ^131^I-tositumomab [5]. A total of 250 patients with heavily pre-treated (median of 4, and up to 13 prior chemotherapy regimens), relapsed or refractory non-Hodgkin lymphoma were included. They received 0.75 Gy to the whole body (or 0.65 Gy if platelet count was 100,000-150,000/mm^3^) based on pre-treatment dosimetry study using a diagnostic amount of 185 MBq ^131^I-tositumomab and 3-timepoint whole-body planar scan (day 0 representing 100% of the activity, with a second scan between day 2 to 4, and a third one between day 5 to 7), to deduce the monoexponential clearance [18]. The therapeutic activity, typically between 2 and 6 GBq, is administered a week later. Immediately before each of the diagnostic and the therapeutic administrations, unlabelled tositumomab (450 mg) is administered to saturate the so-called non-specific binding sites, and this in turn increases the circulation time of ^131^I-tositumomab in the bloodstream, allowing for greater tumour accumulation. Among the 250 patients, any response and a complete response (CR) were seen in 56% (range, 47–68%) and 30% (range, 10–38%) of patients, respectively, and the overall median PFS was 6.4 mo., with 32% of patients achieving a durable response (> 12 mo.). The median durations of any and complete responses were 12.9 and 58.4 mo., respectively. The toxicity was not included in this integrated analysis. In the latter of the five trials, grade 3–4 hematologic and non-hematologic toxicities were seen in 50 and 16% of patients, respectively, while 9% developed hypothyroidism (attributed to the release of ^131^I, and despite thyroid protection with SSKI intake) [6]. In a safety analysis including patients from the five trials, plus 765 others having received ^131^I-tositumomab through an expanded access program, 35 patients (3.5%; 1.6%/year) developed myelodysplastic syndrome (MDS) or acute myeloid leukaemia (AML), among whom the diagnosis was independently confirmed in 23 patients (2.3%; 1.1%/year) [7].

In a subsequent trial of ^131^I-tositumomab RPT in 76 treatment-naïve patients with stage III-IV follicular lymphoma, 95% of patients had an objective response and 75% had a CR [8]. The median PFS was 6.1 years. Forty of the 57 patients with a CR remained in remission after 4.3 to 7.7 years of follow-up. The rates of grade 3–4 adverse events were as follows: neutropenia in 34%, thrombopenia in 17%, and other non-hematologic adverse events in 21%. No patients were subsequently diagnosed with MDS or AML, however. ^131^I-tositumomab radioimmunotherapy thus appeared substantially more effective and better tolerated in treatment-naïve as opposed to heavily pre-treated patients. The subsequent clinical trials of ^131^I-tositumomab were based on a whole-body dose prescription in a non-myeloablative context, and, for some myeloablative protocols, on critical solid organ dosimetry (with autologous stem cell rescue). They are not fully reviewed here.

## ^90^Y-ibritumomab radioimmunotherapy of lymphoma

Contrary to ^131^I-tositumomab, the anti-CD20 radioimmunotherapy with ^90^Y-ibritumomab (Zevalin®) has been approved with a weight-based activity prescription (14.6 MBq/kg). As with the former, there is a pre-treatment imaging study, but without mandated dosimetry. The purpose of the pre-treatment diagnostic imaging with ^111^In-ibritumomab is limited to qualitatively ruling out an altered biodistribution. Like ^131^I-tositumomab, there is a cold antibody infusion – using rituximab in this case – immediately before each of the administrations of the diagnostic and of the therapeutic radiopharmaceuticals, 1 week apart. The rationale for weight-based prescription is related to the short physical half-life of ^90^Y (2.7 days) which becomes the dominant determinant of the effective whole-body half-life because the biological half-life of ^90^Y-ibritumomab is much longer. Consequently, the whole-body dose will be roughly proportional to the injected activity per body weight. Conversely,^131^I-tositumomab has a shorter and variable biological half-life paired with a long physical half-life, resulting in high inter-patient variability in whole-body specific absorbed dose [18]. The weight-based activity administration is valid for the approved, non-myeloablative treatment of follicular lymphoma with ^90^Y-ibritumomab.

However, ^90^Y-ibritumomab has also been studied as a myeloablative treatment in an image dosimetry-guided application. In this more invasive therapeutic approach involving autologous stem cells rescue, sparing the bone marrow and the spleen is not an aim. Rather, the critical dose-limiting organ becomes the liver. In a phase 1, absorbed dose escalation study, 18 patients with relapse or refractory follicular lymphoma who underwent a successful stem cell harvest (following rituximab, cyclophosphamide, and filgrastim) proceeded with a high-dose ^90^Y-ibritumomab radioimmunotherapy. Apart from first three patients who received the standard activity of 14.8 MBq/kg, 15 patients received a personalised activity to deliver 18 Gy (*n* = 5), 24 Gy (6), 28 Gy (3) or 30.5 Gy (1) to the liver. The hybrid pre-treatment dosimetry with ^111^In-ibritumomab consisted in 5-timepoint whole-body planar scan (at < 2, 4, 24, 72, and 144 h) plus a 2-bed SPECT/CT at 24 h to allow the scaling of the time-activity curve. Contrary to what is expected for the whole-body specific absorbed dose with ^90^Y-ibritumomab, there was a large inter-patient variability in the liver specific absorbed dose (median: 2.68, range: 1.21–4.14 MBq/kg/Gy). There was grade 3–4 hematologic toxicity in most cases, as expected. There was not, however, any case of significant hepatic toxicity, suggesting that the liver maximum tolerated dose would be more than 28 Gy, which is supported by the ^90^Y radioembolisation data. Two patients who received ^90^Y-ibritumomab died from disease progression, and another one from pneumonia, while one patient developed MDS. Efficacy results were encouraging, with 16 patients achieving any response, 14 of whom, a CR. Median PFS was 13 mo., but highly variable (range: 0.1–40 mo.), and it was not possible to see any trend for the response rates or the PFS among the small patient cohorts with escalating liver doses. Another example of liver absorbed dose escalation study could be found in a myeloablative strategy against multiple myeloma, with ^90^Y-ibritumomab given as an adjunct to high-dose melphalan [19]. In contrast with the above study, the maximum tolerated liver dose was only 18 Gy, possibly due to a synergistic toxicity with melphalan.

## ^153^Sm-EDTMP RPT of bone metastasis

RPT with bone-seeking radiopharmaceuticals can effectively relieve bone pain in patients with bone metastases that are positive on bone scan. ^153^Sm-ethylenediaminetetramethylene phosphonic acid (EDTMP) is a phosphonate-based bone seeking agent while ^89^Sr chloride, another beta emitter, is a calcium analogue, as is the more recently introduced alpha emitter ^223^Ra chloride. It is noteworthy to highlight that ^223^Ra has been shown to prolong OS in patients with metastatic castration-resistant prostate cancer, but the study was conducted before the introduction of novel anti-androgen drugs [20]. The three radiopharmaceuticals have in common that they were all approved with fixed and/or weight-based activity prescription in their respective package inserts (37 MBq/kg for ^153^Sm-EDTMP; 148 MBq or 1.5–2.2 MBq/kg for ^89^Sr; 55 kBq/kg for ^223^Ra) [21–23]. An imaging dosimetry-based ^153^Sm-EDTMP regime has been developed by the group of Turner et al. from Australia [10]. Initially, they estimated the bone marrow dose using urine collection over 5 hours to deduce whole-body retention, assuming localisation of the retained activity in the bone. To improve the practicality of their approach, they translated the urine-based method to a purely imaging-based one. They reported the results of their pilot study in 10 patients with painful bone metastases (eight with prostate, one with breast, and one with pancreatic cancers). The patients underwent injection of a low activity of 740 MBq ^153^Sm-EDTMP followed by 2-timepoint whole-body planar scans (at 10 min. before miction, and at 5 h after miction) to quantify whole-body activity retention which was assumed to be entirely in the bone, and this was used to compute the bone marrow absorbed dose. The resulting estimates correlated strongly with those derived from urine measurements, and the novel imaging-based method thus appeared as a more practical alternative. The therapeutic injection was done later the same day with a personalised activity to deliver 2 Gy to the bone marrow. There was no hematologic toxicity. Eight patients (80%) responded with complete (60%) or partial (20%) pain relief. The authors compared the bone marrow absorbed dose (1.97–2.07 Gy) with that which would have been delivered with the standard weight-based activity of 37 MBq/kg (3.27–5.90 Gy). Based on this, they extrapolated that the personalised approach may have prevented significant hematologic toxicity. However, in a large, randomised trial of ^153^Sm-EDTMP in which 37 MBq/kg was used, the rates of grade 3 thrombopenia and leukopenia remained modest at 3 and 5%, respectively [24].

## ^131^I-MIBG RPT of neuroendocrine tumours

^131^I-metaiodobenzylguanidine (MIBG), a radiolabelled amine precursor, has been used to image (also with ^123^I-MIBG) and treat pheochromocytoma, paraganglioma, neuroblastoma and some other neuroendocrine tumours such as those from the midgut. Using high-specific activity MIBG, a high-activity regime was studied in a multicentre, single-arm clinical trial in which 68 patients with pheochromocytoma or paraganglioma suffering from secondary hypertension were registered [11]. The regime consisted in 2 cycles of up to 18.5 GBq ^131^I-MIBG. The activity was adjusted per body weight in patients < 62,5 kg (296 MBq/kg), and personalised based on a pre-treatment dosimetry study consisting in the injection of 185 MBq ^131^I-MIBG and 3-timepoint whole-body planar scanning (< 1 h, day 1–2, and day 3–5). There was an activity reduction in cases where pre-treatment dosimetry indicated that the fixed or weight-based activity would predict excessive absorbed dose to the bone marrow (> 12 Gy), lungs (> 16.5 Gy), kidneys (> 18 Gy), liver (> 31 Gy), or small intestine (> 40 Gy), as per the package insert [25]. The choice of these limits is said to be based on the 1991 paper for external radiotherapy by Emami et al., but the latter does not refer to the selected bone marrow dose threshold, so it remains unclear from the study report what motivated the choice of 12 Gy, which is much higher than the more commonly stated 2 Gy limit [11, 26]. Moreover, the proportion of patients in whom the treatment was really de-intensified based on dosimetry was not explicitly reported. Only one case of a patient whose planned cumulative activity was reduced to 3.8 GBq based on dosimetry was given as an example. The fact that the median cumulative activity of 35.7 GBq was very close to the absolute cap of 37 GBq suggests that only a minority of patients underwent significant dosimetry-based personalisation. Eighteen patients (26%) did not receive their second cycle because of hematologic toxicity. This MIBG regime was successful in significantly reducing the anti-hypertensive medication usage (i.e. by at least 50% over 6 mo.) in 25% of patients and as such the primary endpoint of the study was met, leading to the FDA approval in 2018. In 64 evaluable patients, partial response (PR) was seen in 23%, stable disease (SD) in 69%, and progressive disease (PD) in 8%. Biochemical response rates varied between 31% and 68% among the various biomarkers considered, and median OS was 37 mo. There were trends towards greater clinical benefits in patients receiving two rather than one cycle. However, there were high rates of hematologic toxicity for a non-myeloablative treatment, with any-grade hematologic toxicity experienced by 90% of patients and grade 3–4, by 72%. Twenty-five percent of patients required hematologic supportive care. MDS occurred in 3 patients, and AML and acute lymphocytic leukaemia (ALL) in 1 patient each (total of 5 patients, 7%).

## ^177^Lu-DOTATATE PRRT of neuroendocrine tumours

The current empiric fixed-activity regime of ^177^Lu-DOTATATE consists in 4 cycles of 7.4 GBq given at 2-monthly intervals. In their 2008 report, the Rotterdam group retrospectively justified this regime based on their 2001 dosimetry data from only 5 patients: a cumulative activity of 29.6 GBq would result in a bone marrow dose not exceeding 2 Gy [27, 28]. This regimen was then adopted in the NETTER-1 trial, which led to the marketing of ^177^Lu-DOTATATE (Lutathera®) many years later [1]. The NETTER-1 trial was a multicentre international phase 3 trial in which 229 patients with midgut neuroendocrine tumours were randomised to receive ^177^Lu-DOTATATE plus standard dose of long-acting octreotide vs. high dose of long-acting octreotide. The primary endpoint of PFS was met, as ^177^Lu-DOTATATE prolonged PFS by 20 mo., from a median of 8.4 mo. in the control group to 28.4 mo. in the ^177^Lu-DOTATATE group [1]. Toxicity rates were low, with grade 3–4 thrombopenia in 2%, neutropenia in 1%, and renal toxicity in none. The final OS was 48.0 mo. for ^177^Lu-DOTATATE vs. 36.3 mo. for the control group, and although clinically impressive, this difference did not reach the prespecified level of statistical significance [29]. The low toxicity of the empiric regime of ^177^Lu-DOTATATE, combined with the high interpatient variability in renal and bone marrow specific absorbed doses suggest that the majority of patients could tolerate a higher injected activity to increase tumour dose and clinical benefits. Below is summarised the experience of the three groups who reported on dosimetry-based personalised PRRT with ^177^Lu-DOTATATE.

### Uppsala

This group was the first to report results of a prospective clinical trial of personalised PRRT [12]. In a single-arm study, 200 patients with neuroendocrine tumours of mixed origins and grades, and as a last-resort therapy, were administered cycles of a fixed activity of 7.4 GBq ^177^Lu-DOTATATE at 6 to 8-weekly intervals until a cumulative renal absorbed dose of 23 Gy or a bone marrow dose of 2 Gy was achieved. Kidney dosimetry was assessed using SPECT/CT-based dosimetry and 4-cc small-sphere VOI sampling of kidney activity concentration. Scans were performed at 1, 4, and 7 days after the 1^st^ and the 4^th^ administrations, and at 1 day only for other cycles. A mixed blood and whole-body planar scan-based approach was used for the bone marrow dosimetry assessment, but the limit of 2 Gy was not reached in any patient. The prescribed renal dose of 23 Gy was reached in 3 to 9 cycles in 62% of patients, and 49% of patients received more than 4 cycles. Most other patients stopped PRRT because of toxicity or disease progression. Tumour dose was not reported. Overall, the ORR and biochemical response rate were 24 and 67%, and the median PFS and OS were 27 and 43 mo., respectively. Most interestingly, there were statistically significant differences for all these four parameters between the subgroup of patients who have reached 23 Gy to the kidney vs. those who did not: 31% vs. 13% ORR, 80% vs. 45% biochemical response rate, 33 vs. 15 mo. median PFS, 54 vs. 25 mo. median OS. Of note, the 65 patients with midgut neuroendocrine tumour who reached 23 Gy to the kidney had median PFS and OS of 42 and 60 mo., respectively. This compares favourably with the outcomes of the patients in the ^177^Lu-DOTATATE arm of the NETTER-1 trial, at 28 and 42 mo., respectively [30]. While in both studies an identical fixed activity per cycle of 7.4 GBq was given, the number of 2-monthly cycles was variable in the Swedish study. With a median number of cycles of 5, and up to 9 cycles in this subgroup, the induction course of PRRT was thus, for most patients, delivered over a longer period than in the NETTER-1 study.

### Lund/Gothenburg

Final results of another Swedish clinical trial of dosimetry-based PRRT, the ILUMINET trial, were recently reported [13]. In this prospective, single-arm, multicentre, phase 2 clinical trial, 96 patients with mixed gastro-entero-pancreatic NETs were treated with 8 to 10-weekly, 7.4 GBq fixed-activity ^177^Lu-DOTATATE cycles until a renal BED of 27 Gy was reached (which they called *step 1*). Of note, because there is a roughly consistent + 10% difference between absorbed dose and BED for ^177^Lu PRRT, this regime was quite similar to that of the Uppsala group described above, or at most slightly augmented [31]. A subset of 9 patients meeting specific favourable criteria were allowed to pursue the regime until a 40 Gy renal BED was reached (*step 2*). The dosimetry protocol was hybrid, with 5-timepoint whole-body planar (up to day 7) allowing to define the shape of the time-activity curve, which was then scaled using a single-timepoint quantitative SPECT/CT at 24 h. Patients received 1 to 9 cycles (median of 5 cycles) over a period of up to 97 weeks. The primary endpoint was the ORR 3 mo. after the last cycle of *step 1* (i.e. up to 27 Gy renal BED), and the results were as follows: PR in 16%, SD in 66%, and PD in 19% (*n* = 90). When the *step 2* outcomes were also considered, the best ORR reached 2% CR, 32% PR, 61% SD and 4% PD. This illustrates that many patients are late responders to PRRT, although it is surprising that among 17 patients with initial PD, 13 were later found to have SD or better response. The median PFS and OS were 29 and 47 mo., respectively. Both were significantly longer as the delivered renal BED went from < 25 Gy, to 25–29 Gy, to > 29 Gy. The profile of clinical adverse events did not appear to differ from that of other PRRT studies. The laboratory toxicity data showed grade 3–4 rates of 9.4% for thrombocytopenia, 6.2% for neutropenia, 4.2% for leucopoenia, 1% for anaemia, and no grade 3–4 treatment-related renal toxicity.

### Quebec

We are conducting a single-arm trial of personalised PRRT in which patients with mixed neuroendocrine tumours receive four induction cycles of ^177^Lu-DOTATATE, with a personalised activity per cycle to deliver 23 Gy to the kidney. Like the Uppsala group, the renal dosimetry is based on 2-cm VOI sampling using quantitative SPECT/CT. Initially, we performed 3-timepoint imaging (day 0, 1 and 3) at each cycle, and progressively reduced this to 2 timepoints (day 1 and 3) after the first cycle and one timepoint (day 3) after the subsequent cycles [32]. We prescribe a renal dose of 5 Gy at the first cycle (based on body surface area and estimated glomerular filtration rate), and then split the remaining dose to be delivered over the following 3 cycles [33]. Initially, for sake of prudence, we imposed a 50% cap for any activity escalation from one cycle to the next, which we later removed. We reported preliminary results in 52 patients [14]. In 34 patients who completed the induction regime at the time of analysis, the median cumulative activity was 36.1 (range, 6.3–78.6) GBq, and this allowed increasing the tumour dose in 85% of patients, by a median factor of 1.26 fold (up to 2.12 folds), as compared to if they would have been treated with the usual 7.4 GBq fixed activity. In 39 assessable patients, the best objective response was PR 23.1%, SD in 69% (including minor response in 35.9%), and PD in 7.7%. We found the disease control rate (DCR) particularly encouraging in the sub-group with pancreatic neuroendocrine tumours, which tend to be more radiosensitive in general, with 100% of patients achieving at least SD. Median PFS at the time of analysis was 15.9 mo. (based on only 3 patients at risk at that time mark, however) and OS was not reached. We observed grade 3–4 thrombopenia, leucopoenia, neutropenia, and renal toxicity in 5.8, 5.8, 3.8 and 0% of patients, respectively. We observed a higher rate than previously reported of grade 3–4 lymphopenia at 51.9%, with no clinical consequences, however. Interestingly, there was a significant correlation between the absorbed dose to the bone marrow vs. that to the kidney, suggesting that prescribing the latter may contribute to limit excessive bone marrow exposure and hematologic toxicity.

## ^90^Y-DOTATOC PRRT of neuroendocrine tumours

The group from Iowa performed a phase 2 trial of dosimetry-based ^90^Y-DOTATOC PRRT [15]. 25 patients with mixed neuroendocrine tumours received up to 3 cycles of ^90^Y-DOTATOC, with the first one being at a fixed activity of 4.4 GBq (or 1.85 GBq/m^2^ in two paediatric patients) and the activity of the two following cycles was prescribed to reach a maximum of 23 Gy to the kidney or 2 Gy to the bone marrow, with an absolute activity cap of 5.6 GBq. The post-treatment dosimetry protocol included ^90^Y-PET/CT over the kidney at 5 h, to quantify the activity, and 4-timepoint bremsstrahlung SPECT/CT at 6, 24, 48 and 72 h, to determine the bi-exponential kinetics. The bone marrow absorbed dose was derived from blood sampling. They found that in 85% of patients, the administered activity at cycles 2 and 3 differed by more than 20% from the empiric activity. This was due to the highly variable renal specific absorbed dose ranging from 0.32 to 3.0 Gy/GBq. No patients experienced grade 3–4 renal or hematologic toxicity. No efficacy results were reported.

## Combination RPT of neuroendocrine tumours

The group from Iowa also reported on a series of three patients with midgut neuroendocrine tumour who were treated with combined ^90^Y-DOTATOC PRRT and ^131^I-MIBG RPT in a phase 1 trial [16]. These patients underwent pre-treatment dosimetry assessment using ^111^In-octreotide (as a surrogate for ^90^Y-DOTATOC) and a diagnostic amount of ^131^I-MIBG, with double-isotope planar and SPECT/CT imaging and blood sampling at 1, 4, 24 and 48 h. They determined the dosimetry for the kidneys, target tumour lesions and the bone marrow (self-dose from blood counting and cross-dose from imaging), and prescribed the therapeutic activities of ^90^Y-DOTATOC and ^131^I-MIBG to deliver total cumulative absorbed doses targets of 19 Gy to the kidney and 1.5 Gy to the bone marrow, divided in 2 cycles. As compared with administering personalised ^90^Y-DOTATOC alone, they could slightly reduce the ^90^Y-DOTATOC activity prescription (from 5–10.8 to 2.8–8.7 GBq) to infuse 11.4–18.7 GBq ^131^I-MIBG the next day, with resulting per-lesion dose increases ranging from 34 to 362%. All subjects had stable disease at 6 mo. after cycle 2. One subject experienced temporary grade 3 thrombopenia and another one grade 2 renal toxicity. The authors initially planned for a 3 + 3 absorbed dose escalation study but interrupted it because of logistical challenges and their interest into switching to a combination of ^177^Lu-DOTATATE and high specific activity MIBG (SPORE-3 trial; NCT04614766).

## RPTs of particular interest without prospective dosimetry-guided experience

Above, we reviewed published results of prospective studies in which imaging-based dosimetry has been used to personalise RPT. It is noteworthy that for ^131^I of thyroid cancer, the oldest RPT that is still commonly practised worldwide, as well as for ^177^Lu-PSMA-RLT of prostate cancer, the new blockbuster RPT, prospective data for dosimetry-driven regimes is not yet available.

### ^131^I of thyroid cancer

^131^I has been administered for about 80 years to patients with differentiated thyroid cancer. The most common practice is to administer lower fixed activities in the context of thyroid remnant ablation (e.g. 1.1 to 3.7 GBq), or higher fixed activity to treat patients with documented metastatic disease or at higher risk thereof after surgery (e.g. 5.6 to 7.4 GBq). An imaging dosimetry-based approach has been often cited, i.e. that of Benua and Leeper, in which absorbed dose limits to the bone marrow (2 Gy) and to the lung (30 Gy) are imposed. These limits have been deemed equivalent to a whole-body activity retention on the 48 h whole-body planar scan of 4.4 or 3.0 GBq, in the absence or presence of diffuse lung metastases, respectively [34, 35]. Prospective results using this or other approaches have not been reported yet, to our knowledge. There is however at least one ongoing clinical trial using ^124^I-PET/CT for pre-treatment dosimetry to adjust the ^131^I activity to deliver 80 Gy to the soft tissue lesions and 650 Gy to the bone lesions, without exceeding 2 Gy to the bone marrow as estimated by blood sampling (NCT05299437).

### ^177^Lu-PSMA-RLT of prostate cancer

PSMA-RLT has rapidly emerged over the last decade. With the positive results of the VISION trial showing prolonged PFS and OS following ^177^Lu-PSMA-617 as compared to best supportive care, PSMA-RLT is poised to soon become the most prominent RPT in terms of patient numbers [2]. The Australian study TheraP also showed superior biochemical response rate and PFS, and less toxicity with ^177^Lu-PSMA-617 vs. cabazitaxel chemotherapy [3]. As per the VISION trial, and without explicit dosimetry rationale, the empiric fixed-activity regime for ^177^Lu-PSMA-617 has been established to six 6-weekly cycles of 7.4 GBq. No prospective results and no entry on ClinicalTrials.gov were found regarding dosimetry-based PSMA-RLT. Nevertheless, the rationale for dosimetry-driven PSMA-RLT is the same as for other RPTs: there is a high interpatient variability in specific absorbed dose to critical organs (bone marrow, kidneys, salivary glands) and the current fixed-activity regimes are well tolerated, implying that a majority of patients are undertreated from a dosimetry perspective [2, 3, 36]. Furthermore, a clear tumour dose-response relationship in the context of PSMA-RLT has been evidenced by Violet et al. Among 30 patients treated with fixed-activity ^177^Lu-PSMA-617, those who biochemically responded (i.e. prostate-specific antigen decline > 50%) received a median mean tumour absorbed dose of 14.1 Gy, as compared with 9.6 Gy for those who did not [36]. In addition, negative correlations between the specific absorbed dose to the parotids or the kidneys and the volume of disease were observed, showcasing a tumour sink effect by virtue of which significant radiopharmaceutical sequestration by the tumour translates into reduced exposure of healthy tissues. This implies that in a dosimetry-based regime, patients with a large tumour burden could benefit from further increase in injected activity.

## Discussion

The cumulative evidence drawn from the studies presented here, covering a variety of therapeutic beta-emitting radiopharmaceuticals used in a range of different oncological situations, as well as varied dosimetry approaches (including both pre-treatment and per-treatment dosimetry) broadly illustrates that personalised RPT based on dosimetry (1) is feasible, (2) is safe, and (3) offers the prospect of improved clinical outcomes vs. fixed-activity empiric regimes.

### Feasibility

A critical aspect for wide adoption of any dosimetry-based RPT regime is its feasibility in routine clinical practice. While the required SPECT/CT and/or PET/CT equipment is readily available in most centres offering RPT, a significant logistical hurdle that may arise for many centres is the limited technologist workforce to perform multi-timepoint scanning for a large number of treated patients on top of the usual diagnostic imaging workload. Solutions include the rationalisation of the number of imaging timepoints. Recently, there has been a growing interest in one-timepoint scanning protocols, in which a carefully chosen scanning timepoint (e.g. within a certain time window relative to the population effective half-life for the tissue of interest) would allow to derive a dosimetry estimate with a reasonably small uncertainty [37, 38]. However, outlier patients may handle radiopharmaceuticals with kinetics that significantly deviate from those of the population, and in such cases there will be a systematic underestimation of the absorbed dose, potentially leading to a therapeutic overdosage if that estimate is used to personalise the treatment [39]. A balance between practicality and accuracy is thus essential, and one approach towards this, applicable to multicycle RPT regimes, is to obtain two or three scanning timepoints at the first cycle, then one timepoint at subsequent cycles, in order to gather patient-specific half-lives to be applied to subsequent cycles [32]. Another aspect that can improve efficiency is to use only quantitative SPECT/CT for organ dosimetry, which is more accurate than planar imaging, and requires less effort to process, particularly when simplified activity concentration sampling techniques are used (e.g. small-sphere VOIs for the kidney dosimetry) [12, 32]. Optimising imaging protocols also benefits the patient, with increased comfort and convenience.

Another facet of feasibility is the question of performing full pre-treatment dosimetry using either a surrogate long-lived diagnostic tracer (labelled with, e.g., ^86^Y, ^89^Zr, ^64^Cu, ^124^I for PET, or ^123^I, ^111^In for SPECT) or a low activity of the therapeutic compound, versus performing only post-treatment dosimetry to personalise all but the first cycle based on dosimetry measurements. The former approach is obviously essential in the case of a single-cycle regime. While there is a risk that the low-dose tumour irradiation during the pre-treatment dosimetry induces radioresistance (a.k.a. stunning), the main advantage is to deliver a first (or single) cycle during which tumour irradiation will be maximised, potentially leading to enhanced cytotoxicity among radiation-naïve tumour cells (i.e. before these acquire significant radioresistance). In the case of a multi-cycle regime, however, performing a full pre-treatment dosimetry performed over multiple days adds to the logistical and patient burdens and can significantly delay the initiation of the treatment. Further data will be required to find out if the therapeutic benefits would outweigh the disadvantages of the extra diagnostic procedure in this setting.

Alpha particle RPT is rapidly emerging and is seen by many as the future of RPT. Despite the technical difficulties to image alpha radionuclides owing to the low activities involved (about three orders of magnitude less that in beta RPT) and the unfavourable emission spectrum for many of these, personalised RPT is still achievable. Long-lived PET or SPECT surrogate diagnostic tracers can be used for pre-treatment dosimetry, or co-injected with the alpha-emitting radiopharmaceutical for per-treatment dosimetry. Co-injection of alpha and beta radiotherapeutics having the same pharmaceutical moiety is another approach to consider: the beta emitter could synergise with the alpha emitter by improving crossfire capability for better coverage of heterogeneous lesions, while also enabling high-quality imaging for real-time dosimetry and response assessment.

### Safety

RPT is, in general, reputed for its excellent safety profile as compared to other oncological treatments such as chemotherapy. This is particularly true for ^177^Lu-based PRRT and PSMA-RLT when administered at fixed activity [1, 2, 27]. The clinical results presented above tend to show that, as expected, personalised PRRT does not compromise the overall tolerance, while allowing for a substantial tumour radiation boost in many patients. This also indicates that the safety dose limits derived from external radiotherapy are likely too conservative for RPT, and this clearly opens the door to further absorbed dose escalation. But attempting intensification by further escalating an empiric fixed activity would be hazardous considering the huge inter-patient variability in specific absorbed doses, and would thus predictably result in increased toxicity rates. Indeed, in the high-activity ^131^I-MIBG trial presented above, the treatment was not truly personalised for most patients who received the maximum (or close to) 18.5 GBq/cycle, and this has led to 26% of patients not being able to receive the second cycle because of hematologic toxicity, possibly depriving them of more sustained clinical benefits [11].. The aggregated toxicity data from ^177^Lu and high-activity MIBG RPTs also supports the hypothesis of a certain proportionality, at the population level, between the risks of deterministic hematologic effects (subacute myelosuppression) vs. the stochastic effects (e.g. MDS/AML): approximately 10% vs. 1%, respectively, for ^177^Lu, and 70% vs. 7%, respectively, for high-activity MIBG. Furthermore, the non-myeloablative radioimmunotherapy data falls somewhere in between. Accordingly, the subacute hematologic toxicity rate of a given RPT regime is likely predictive of the incidence of longer-term complication rate. Fractionation can be an important aspect to consider when establishing an RTP regime, and it is possible that more frequent, lower-activity MIBG cycles may be easier to tolerate, while allowing for equal or even greater efficacy if interruption for toxicity can be avoided. For RPT, as for any anticancer treatment, seeking an appropriate balance between risks and potential benefits remains of paramount importance.

### Improved outcomes

The superior efficacy of ^90^Y radioembolisation based on tumour absorbed dose prescription over that for the standard regime has been demonstrated with a high level of evidence, i.e. in a multicentre randomised controlled trial [4]. Such demonstration is yet to be made for systemic RPTs, for which the dose prescription needs to take into account more radiosensitive critical tissues to spare (e.g. the bone marrow and the kidney). At least, it has been shown that personalisation based on healthy organs allows to safely increase tumour dose in most patients, as demonstrated in three independent single-arm clinical trials of personalised ^177^Lu PRRT [12–14].. The response rates and survival figures are difficult to compare with those from historical cohorts because the patients’ and tumours’ characteristics likely differ. Whether ORR, PFS or OS can be improved with dosimetry will require direct comparison in randomised trials against established fixed activity regimes. Once this milestone is achieved for at least one prominent systemic RPT (e.g. ^177^Lu PRRT or PSMA-RLT), it can be foreseen that future development of new systemic RPTs will be dosimetry-based from the start. But until then, the reference standard of practice will remain that of fixed activity, which has prevailed since the introduction of ^131^I for thyroid cancer until today, with the recent approval of empiric ^177^Lu-PSMA-617 RLT for prostate cancer [2].

Another aspect worth considering in the design of a personalised RPT regime is the biological behaviour of the targeted tumour. For example, more indolent cancers such as well-differentiated neuroendocrine tumours may benefit from prolonged exposure to PRRT. Indeed, in the two Swedish studies presented above, the PFS and OS tended to be longer for patients who received the prescribed renal dose vs. those who did not. Because of the fixed activity per cycle of 7.4 GBq, most patients underwent more than 4 cycles, meaning that their PRRT extended beyond 6 mo. (i.e. three 2-monthly intervals in a standard four-cycle induction course). Further substantiating this hypothesis are the results from a Canadian phase 2 trial of low-activity ^177^Lu-DOTATATE consisting in four 3-monthly induction cycles of up to 5.55 GBq, followed by eight 6-monthly maintenance cycles of up to 3.7 GBq. While the median PFS in their cohort of mixed neuroendocrine tumours was 36.1 mo., that in the subgroup of patients with midgut tumours was impressive at 47.7 mo [40]. Further optimising such a maintenance PRT regime by making it dosimetry-based (i.e. low absorbed dose to critical organs per cycle) could be the key for prolonged disease control over many years for patients with indolent tumours. Conversely, escalating treatment intensity with higher absorbed doses per cycle and shorter intervals may be best for more aggressive cancers such as high-grade neuroendocrine tumours or advanced prostate cancer. Again, the balance between risks and potential benefits must be adjusted for the clinical situation of each individual patient in personalised care.

## Conclusion

The current decade will undoubtedly see a tremendous growth in the field of RPT. There has never been a better opportunity to modernise the practice of RPT and make it on par with that of external radiotherapy: personalised and dosimetry-guided. RPT has a unique advantage above ordinary drugs, as it enables non-invasive assessment of the biokinetics of its active ingredient – i.e., dosimetry – at the patient level. While there is still much to learn on how to best deliver RPT, the cumulative evidence to date fully supports the acceleration of clinical research initiatives in dosimetry-based RPT. An eloquent demonstration has been made that personalised radioembolisation is a better treatment than the conservative standard approach, and this now needs to be evidenced for systemic RPTs. Finally, dosimetry-guided RPT represents a more equitable approach for the diversity of patients we treat, because it allows each of them to receive an equivalent treatment from a radiation delivery perspective which is a clear theoretical benefit over fixed activity RPT.

## Data Availability

Not applicable.

## Abbreviations

ALL: acute lymphocytic leukaemia
AML: acute myeloid leukaemia
BED: biologically effective dose
CR: complete response
CT: computed tomography
DCR: disease control rate
DOTATATE: DOTA-DPhe^1^-Tyr^3^-octreotate
DOTATOC: DOTA-DPhe^1^-Tyr^3^-octreotide
EDTMP: ethylenediaminetetramethylene phosphonic acid
MDS: myelodysplastic syndrome
MIBG: metaiodobenzylguanidine
ORR: objective response rate
OS: overall survival
PFS: progression-free survival
PD: progressive disease
PET: positron emission tomography
PR: partial response
PRRT: peptide receptor radionuclide therapy
PSMA-RLT: prostate-specific membrane antigen radioligand therapy
RPT: radiopharmaceutical therapy
SD: stable disease
SPECT: single-photon emission computed tomography
VOI: volume of interest
